# Microbial Nanoculture as an Artificial Microniche

**DOI:** 10.1038/srep30578

**Published:** 2016-08-01

**Authors:** Tagbo H. R. Niepa, Likai Hou, Hongyuan Jiang, Mark Goulian, Hyun Koo, Kathleen J. Stebe, Daeyeon Lee

**Affiliations:** 1 Department of Chemical and Biomolecular Engineering, University of Pennsylvania, Philadelphia, PA 19104, USA; 2 School of Mechatronics Engineering, Harbin Institute of Technology, Harbin 150001, China; 3 Department of Biology, University of Pennsylvania, Philadelphia, PA 19104, USA; 4 Department of Orthodontics, Pediatric Dentistry and Community Oral Health Divisions, University of Pennsylvania, PA19104, USA

## Abstract

Microbes self-organize in microcolonies while transitioning to a sessile form within a protective biofilm matrix. To enable the detailed study of microbial dynamics within these microcolonies, new sessile culture systems are needed that sequester cells and mimic their complex growth conditions and interactions. We present a new nanoliter-scale sessile culture system that is easily implemented via microfluidics-enabled fabrication. Hundreds of thousands of these nanocultures can be easily generated and imaged using conventional or confocal microscopy. Each nanoculture begins as a several nanoliter droplet of suspended cells, encapsulated by a polydimethylsiloxane (PDMS) membrane. The PDMS shell provides long-lasting mechanical support, enabling long term study, and is selectively permeable to small molecules including antibiotics, signaling molecules and functional fluorescent probes. Thus, as microcolonies mature within the nanocultures, they can be stressed or interrogated using selected probes to characterize cell physiological properties, antibiotic susceptibilities, and antagonistic interactions. We demonstrate this platform by investigating broad ranges of microcolony dynamics, including direct and indirect bacterial-fungal interactions. This versatile new tool has broad potential for addressing biological questions associated with drug resistance, chronic infections, microbiome dynamics, and antibiotic discovery.

Microbes play diverse roles, as infectious and therapeutic agents, as producers of toxins and pharmaceuticals, as foulants and remediators, and others that we continue to unveil, both beneficial and deleterious, in nature, human health^1^, and industrial applications^2^. As communal interactions are key to their survival, microbes typically self-organize in microcolonies, precursors to biofilms, comprising aggregates of sessile cells that secrete polysaccharides, enzymes, and other molecules to communicate and to form a protective matrix. Within these aggregates, physical and chemical heterogeneities modulate local intercellular interaction and growth, and spatial compartmentalization allows physicochemical challenges to develop associated with changes in pH^3^, and gradients in nutrients and chemicals^4^. The microevolution and the adaptability of cells within such microniches, and their subsequent maturation into biofilms are implicated in biofouling, and in persistent infection^5^,^6^,^7^,^8^,^9^. In cystic fibrosis, for instance, microbes elude the immune system and resist antibiotic treatment^10^ via the formation of microcolonies in the alveoli^11^ to establish a diverse and resilient ecosystem^12^,^13^. In this environment, cells trade their planktonic (free-swimming) lifestyle for restricted but safe confinement in starving biofilm communities surrounded by phagocytic cells. There, they engage in cooperative or competitive interactions to acquire new antibiotic resistance through physical, chemical and genetic interactions.

In spite of their importance, there is a paucity of information on microbial dynamics within these microcolonies. To develop this field, new sessile culture systems are needed that mimic the complex growth conditions and physico-chemical interactions in these microenvironments. An ideal system would be easily implemented at low cost from readily available materials to provide reproducible conditions within a microniche. Such a platform could be used to investigate broad ranges of microcolony dynamics, including microbial pathophysiology and inter-species interactions, and could pave the way to new approaches to control their microevolution more effectively. If the platform also allowed communication with its internal and external environments by permitting nutrient or chemical fluxes, biological questions like those associated with interactions of adjacent cells within the microcolonies^4^,^14^, and finite concentration of small molecules, could be addressed^15^. Such studies could help decipher the complex nature of the biofilms^16^, and explain how community behavior can be modified by subverting the communication patterns of cells or changing the surrounding milieu, e.g. by forcing them to sense signaling molecules under physical confinement^17^. Furthermore, such systems would provide new avenues to explore, within a controlled space, cross-kingdom microbial interactions and inter-species dynamics, which are dependent on time and growth-phase, and which are critical to find new bioactive metabolites.

Confined cell culture systems that address some of these needs have recently been developed. Diffusion growth chambers are one example; such systems successfully contain microorganisms within a physical barrier, while sustaining nutrient availability and transport of secondary metabolites. One such system, the iChip, has had remarkable success in the screening for new bioactive molecules following the growth of challenging microbial species *in situ*^18^. However, this system has some limitations. Inoculation can be time consuming and expensive. It requires micromachining of multiple chambers (∼200 for iChips) to isolate cells, and allows only a single growth condition to be studied for each microfabricated platform. A second approach, based on microfluidics, generates water-in-oil-in-water (W/O/W) emulsions to encapsulate, isolate and culture bacteria cells^19^; this is an inexpensive, easy and rapid means of isolating cells in multiple microcompartments (300,000 capsules in 10 min). However, the fragility of the emulsion droplets and limited understanding of mass transfer in these systems pose challenges for robust, long-term studies of cell growth.

Inspired by these studies, we have developed nanoliter-scale growth chambers that combine some of the advantages of both systems into a multifunctional microbial nanoculture system. Using a microfluidics method to control the amount of inoculum, and the size and the generation rate of the nanocultures, we grow microcolonies with variable cell density in a highly reproducible manner. We use polydimethylsiloxane (PDMS), an inexpensive non-toxic material, to form a robust membrane surrounding a cell suspension of 0.5–5 nanoliter; because water can permeate across the PDMS shell, the volume of the nanoculture can be further manipulated via osmotic stress. The robustness of the shell allows these compartments to be exposed to shaking/stirring conditions, which is typically necessary to facilitate gas transfer. In addition, The PDMS-based nanoculture exhibits selective permeability to various low molecular species including antibiotics, fluorescent dyes, and signaling biomolecules. We use the selective permeability as a feature to spur or alter microbial growth via the addition bioactive molecules in the external phase. Thus, we introduce a multifunctional, tunable nanoculture system to encapsulate and grow bacteria and fungi (in single or co-culture models), and to interrogate the cells with specific molecules. We illustrate the versatility of this platform in the study of complex microbial growth dynamics, as well as in inter-kingdom interactions within compartmentalized microenvironments, which could ultimately lead to the discovery of new bioactive molecules.

## Results and Discussion

### Characterization of PDMS Nanoculture systems

**Figure 1:**
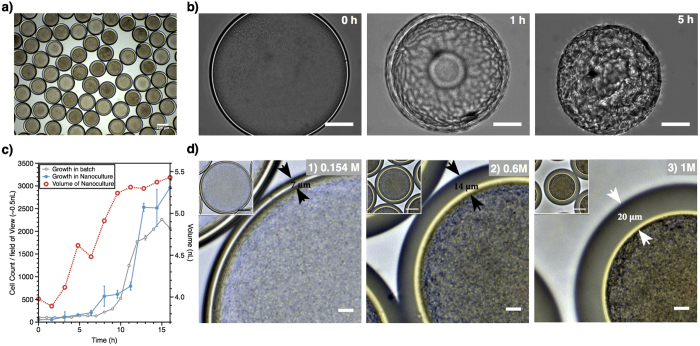
*Pseudomonas aeruginosa* in the PDMS nanocultures. (**a**) The cells were encapsulated in the nanocultures containing UFTYE medium and incubated at 37 °C for 24 h. (**b**) Osmotic annealing of PDMS after curing. The PDMS nanocultures were cured by incubating at 37 °C for 24 h, conferring mechanical robustness to withstand osmotic annealing in 10% NaCl solution (scale bar: 50 μm). (**c**) The growth kinetics of *P. aeruginosa* PA14 in PDMS nanoculture. Nanocultures containing PA14 cells in UFTYE medium were collected in 154 mM NaCl solution and cell growth was monitored at 37 °C for 16 h using a microscope. The volume of each culture was ∼4 nL. Growth in the nanocultures correlated with an increase in size of the nanocultures from 4 to 5.4 nL. For comparison, the cell count for the PA14 grown in a shaken flask is also presented (batch). The data were scaled down to a volume of 0.5 nL, representing a field of view with a radius of 103.5 μm (average radius of the nanoculture) and height of 15 μm (cell+flagellum). Standard deviations are shown. (**d**) Osmotic annealing of the nanoculture during PDMS curing. The nanocultures of PA14 cells in UFTYE medium collected and incubated in solutions with increasing NaCl concentration (0.154, 0.6 and 1 M). Water diffusion out of the nanocultures occurred during the polymerization of PDMS, resulting in the thickening of the PDMS shell (7, 14, and 20 μm for nanocultures in 0.154, 0.6 and 1 M NaCl, respectively) and in higher confluence of the encapsulated cells (scale bar: 10 μm; inset scale bar: 50 μm).

PDMS nanocultures were generated at a frequency as high as 235 Hz using a glass capillary microfluidic device (Supplementary Fig. S1). The W/O/W double emulsions were collected in a saline solution containing 154 mM NaCl, and incubated at 37 °C for 24 h to induce crosslinking of PDMS, in the presence or the absence of 5% CO_2_ according to the cell growth requirements. This procedure results in the formation of capsules with nanoliter chambers; we call each of these capsules a *nanoculture* (Fig. 1a). PDMS was selected because this biocompatible polymer can be cured to form mechanically robust membranes that withstand manipulation without breaking. More importantly, PDMS is known to be permeable to water as well as small molecules, which will play a crucial role in the functionality of these nanocultures^20^,^21^. As shown in Fig. 1b, the imposition of a strong osmotic stress (10% NaCl) for 5 h around nanocultures that had been polymerized after 24 h of incubation at 37 °C resulted in wrinkling and collapse of the PDMS nanocultures (Supplementary Video S1) without the loss of cells, indicating that the crosslinked PDMS shell is water-permeable, pliable and mechanically robust.

Growth kinetics of model organisms including *Pseudomonas aeruginosa* and *Escherichia coli* (bacteria), and *Candida albicans* (fungus) were investigated in the PDMS nanocultures. For instance, Fig. 1c shows that *P. aeruginosa* PA14 cells were successfully grown overnight in nanocultures containing ultrafiltered tryptone-yeast extract broth (UFTYE, low molecular weight <10 kDa). Real-time monitoring of PA14 cells within the PDMS nanoculture demonstrated that the exponential growth phase was reached between 6 h–14 h of incubation (Fig. 1c, Supplementary Video S2), followed by the stationary stage. This trend was very similar to the growth of PA14 in a normal culture. Cell growth within the nanoculture also corroborated with the increased volume of the nanocultures (from 4–5.2 nL), which we attributed to the generation and accumulation of free metabolites, and the associated osmotic pressure gradient across the membrane that drives swelling of the core by water permeation prior to the complete curing of the PDMS shell.

This ability of water to permeate the PDMS shell is essential to tune the microniche dynamically. For example, osmotic pressure differences between exterior and interior can be adjusted to control cell confluence within the nanoculture. Incubation of the microniches in solutions with increasing salinity (154, 600, and 1000 mM NaCl) resulted in osmotic pressure-induced shrinking of the nanocultures, and concomitant increased cell confluence (Fig. 1d1). The osmotic annealing also caused the shell thickness to increase (from 7 to 20 μm), likely enhancing the mechanical durability of the shell, and making the microniche stable for long-term studies.

To test the robustness and durability of the nanoculture system, we evaluated the persistence of *P. aeruginosa* PA14 Δ*pelA* in the nanoculture for several months. *P. aeruginosa* can survive under nutrient-deficient conditions for a period of 12 months^22^. PA14 Δ*pelA* was chosen for its lack of PelA expression, a major polysaccharide of the PA14 biofilm matrix that also accounts for the initial attachment and aggregation of individual bacterial cells^23^. Preventing the formation of the biofilm matrix facilitates tracking the motion of viable cells within the nanocultures. The cells indeed persisted in the PDMS nanocultures, and remained viable under nutrient limitation over a period of 7 months (Supplementary Video S3). Meanwhile, the nanocultures remained intact, demonstrating that the robustness and durability of our system could enable the cultivation of microorganisms in their natural environment and/or advance the systematic understanding of starvation and bacterial persistence by examining phenotypic changes of the cell.

### Growth control through semi-permeability of PDMS nanoculture system

While water permeation is a powerful tool, the ability to introduce small molecules such as fluorescent probes and antibiotics into the cell growth chamber would expand the utility of the nanoculture system. For instance, many bacteria are known to secrete small molecules to spur biofilm growth^24^. If such molecules can be transported across the PDMS membrane, the growth dynamics of cells residing in the nanoculture could be influenced by another community in the external phase; such studies could enable the discovery of new bioactive molecules capable of promoting or impeding cell growth.

**Figure 2:**
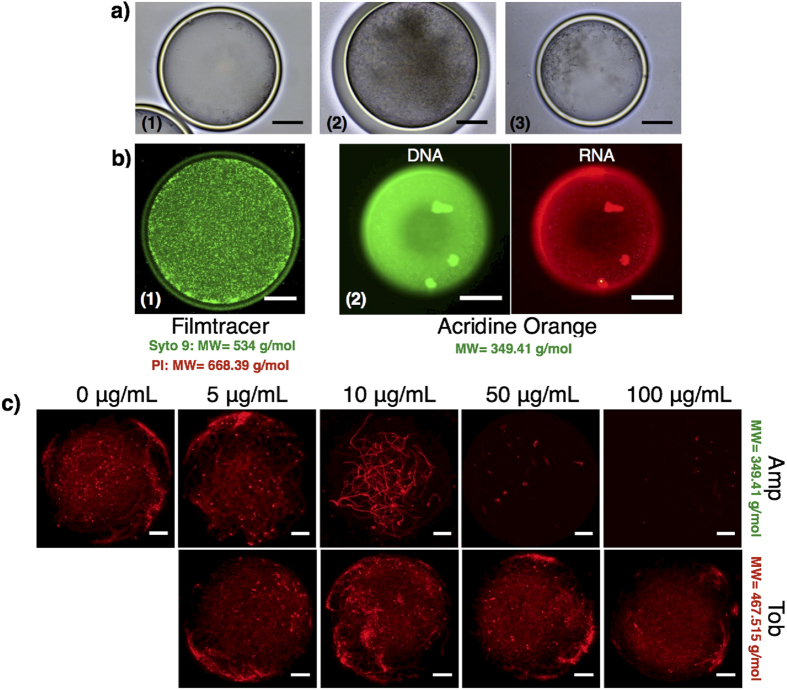
Diffusion of small molecules including signaling molecules and fatty acids into PDMS nanoculture could promote cell growth. Nanocultures of *E. coli* RP437 (**a1**) were incubated at 37 °C for 24 h in a supernatant solution (**a2**) collected from a *E. coli* RP437 overnight culture, which resulted in further increase of cell density within the nanocultures. Growth in the presence of supernatant solution was much significant compared to when the nanoculture were resuspended in sterile growth medium only (**a3**). (**b1**) Staining of PDMS nanoculture systems with Filmtracer™ LIVE⁄DEAD^®^ Biofilm Viability Kit revealed PDMS nanocultures are permeable to Syto 9 but not to Propidium Iodide. (**b2**) Staining of PDMS nanoculture with acridine orange is a complementary approach to assess cell vitality within the nanocultures. PAO1 cells were successfully stained with acridine orange to report metabolic activity through RNA expression in a 24 h nanoculture. (**c**) PDMS nanocultures were also permeable to antibiotic Amp. The antibiotic inhibited the growth of the *E. coli* RP437 cells during the 24 h incubation period. The inability of Tob to cross PDMS nanoculture and to kill bacterial cells suggested both chemistry and size-based selectivity of the nanoculture system.

To investigate this concept, we tested whether cells growing inside the nanoculture could be affected by the presence of small molecules outside the capsule. *E. coli* RP437 cells were encapsulated in the nanocultures, incubated in 154 mM NaCl solution, and grown overnight to reach stationary stage at 37 °C (Fig. 2a1). The nanocultures were then transferred to a supernatant from an overnight culture made with the same strain grown in a 250 mL flask. The result, shown in Fig. 2a2, clearly shows that the addition of the supernatant in the external environment of the nanocultures promoted cell growth compared to the control with no supplementation. To confirm that the resulting cell confluence could not be attributed to water outflow via osmotic stresses, the same experiment was repeated, with the sole exception that the nanocultures were re-inoculated in sterile growth medium only, i.e. without signaling molecules, metabolites or waste products. No significant enhancement in cell density was observed (Fig. 2a3), suggesting the increase in the cell density was indeed associated with the flux of bioactive molecules across the PDMS shell.

The utility of the nanoculture system would be further enhanced if one could assess other features like cell activity and cell vitality within the microcolonies using appropriate fluorescent probes. We demonstrate this capability by culturing *P. aeruginosa* PAO1 cells in the nanoculture, and adding different functional dyes to the external phase. PAO1 cells were successfully stained after re-suspending the 24 h-nanocultures in a solution containing 1.5 μL/mL Syto 9, a green fluorescent nucleic acid dye (MW = 534.44 g/mol). The staining allowed the visualization of PAO1 accumulation at the inner wall of the nanocultures (Fig. 2b1) as well as a large number of cells that remained active and motile within the nanocultures after incubation for 24 h (Supplementary Video V4). Similarly, acridine orange, another fluorescent probe that emits a green or red fluorescence based on the interaction with double stranded DNA or single stranded RNA expressed by the PAO1 cells, respectively, was successful in staining PAO1 cells within the nanocultures after 24 h incubation (Fig. 2b2). We then characterized cell viability by means of a third functional probe, propidium iodide (PI) that detects dead cells. Surprisingly, the dead cells were detected only when 1.5 μL/mL PI was introduced in the original cell suspension during encapsulation, instead of to the external phase (PI, MW = 668.39 g/mol). These results suggest that the PI does not cross the shell of the nanoculture, which we attribute to the low solubility of PI (a charged fluorescent dye) in PDMS^25^.

Another potential significant application of the nanoculture would be their exploitation as tools for high throughput screening for new antibiotics. The high frequency and reproducible generation of the nanoculture could facilitate the encapsulation, isolation and growth of multiple unculturable bacterial species, thought to secrete bioactive molecules with antimicrobial properties. The robustness and durability of the nanocultures could enable incubation of the microniches in their original environments (e.g. soil load, sea water). The nanoculture could be introduced in cultures of pathogenic strains to directly characterize the antimicrobial compounds secreted by competing species, especially if we can control the selectivity of the nanocultures. Furthermore, the nanoculture system could facilitate the study of multidrug resistance through screening of a library of mutants exposed to various antibiotics. However, to enable these discoveries, it is critical to understand whether antibiotics can transport through the PDMS shell and to understand what factors determine their permeability in PDMS.

We tested permeability of the nanoculture shell to various antibiotics by monitoring antibiotic susceptibility. Four classes of antibiotics, including β-lactam ampicillin (Amp), aminoglycoside tobramycin (Tob), fluoroquinolone ciprofloxacin (Cip), and tetracycline (Tet), known to have various killing mechanisms^26^, were tested on *E. coli* RP437 cells. All of these antibiotics exhibit strong inhibitory activities against *E. coli* RP437 cells when the cells are directly exposed to the drugs (Supplementary Fig. S2a). *E. coli* RP437 expressing a red fluorescent protein (RFP) was encapsulated in PDMS nanocultures and incubated at 37 °C for 24 h under different antibiotic concentrations. The results in Fig. 2c clearly demonstrate that the growth of RFP-expressing *E. coli* was affected by 50 μg/mL Amp and 5 μg/mL Cip, proving that the β-lactam Amp and the fluoroquinolone Cip crossed the PDMS shell. In contrast, the aminoglycoside Tob exhibited limited transport through the PDMS shell, as no growth inhibition was observed. Also, Tet (tetracylcine) was impermeable to the PDMS nanoculture. The inability of Tob (aminoglycoside) and Tet to permeate through the PDMS shell was also confirmed on nanocultures of *P. aeruginosa* PA14 cells, as the PA14 cells proliferated in the nanocultures incubated in the presence of 100 μg/mL Tet or 100 μg/mL Tob^27^,^28^ (Supplementary Fig. S2b).

Our first speculation was that the molecular weight of the compounds might be a factor in dictating their flux through the PDMS shell. The permeable antibiotics, Cip and Amp, have a molecular weight of 331.42 and 349.42 g/mol, respectively, smaller than 444.43 and 467.51 g/mol, the molecular weights of the impermeable antibiotics Tet and Tob, respectively. However, we have detected the transport of molecules that have molecular weight as large as 700 g/mol (Supplementary Fig. S3) and also observed that small molecules such as glucose and sucrose do not cross the shell, suggesting that the permeability of the nanocultures is not trivially linked to the molecular weight of the molecules. Because permeation through PDMS involves the partitioning of a molecule from the external phase to the polymer phase, it is highly plausible that the solubility of the solute in PDMS is an important factor. While the partition coefficient of each molecule between water and PDMS would provide a quantitative description of such solubility^29^, such information is not readily available. As a proxy for solubility, we calculate the Flory–Huggins interaction parameter (χ), a measure of the magnitude of enthalpic interactions between the repeat unit of PDMS and each antibiotic or solute^30^,^31^ (See Supplementary Information for detailed calculations of χ).

**Figure 3:**
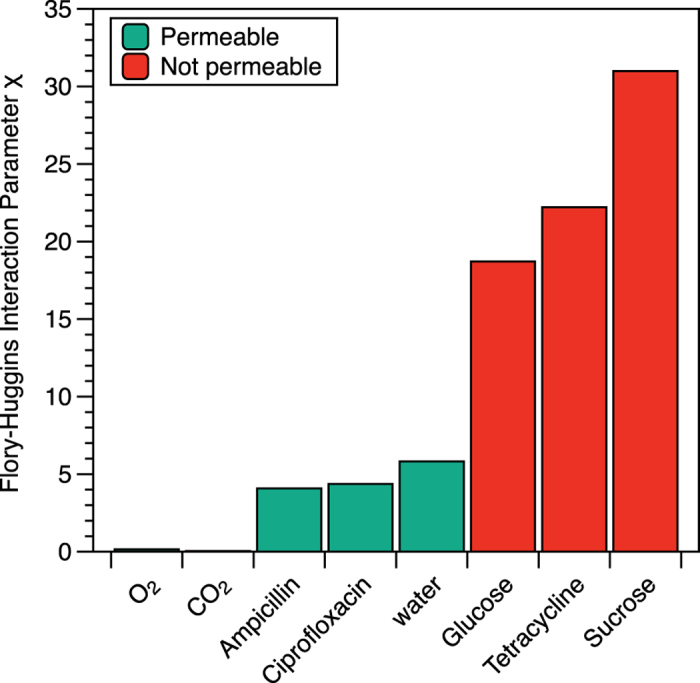
Flory-Huggins interaction parameter between PDMS and several molecules demonstrates the existence of a threshold χ value for molecules crossing the shell of the nanocultures.

Our results (Fig. 3) demonstrate that the miscibility of PDMS with Amp (χ=4.08), Cip(χ = 4.37), and water (χ = 5.83) are significantly greater than that of glucose (χ = 18.72), Tet (χ = 22.22), and sucrose (χ = 30.99). Glucose, Tet and sucrose have very large interaction parameters, suggesting that they do not mix well with PDMS, and therefore are essentially impermeable in PDMS. These results point to the importance of understanding the interactions between the material that forms the physical barrier for the nanoculture and the permeant by, for example, varying the composition of the shell to tailor the selective permeability of the nanoculture system. While we cannot estimate the Flory-Huggins parameter between PDMS and PI (the dye that did not cross the PDMS shell), we suspect that the low solubility of PI may be attributed to its unfavorable enthalpic interactions with PDMS.

### Assessing antagonistic inter-kingdom interaction in nanoculture system

The nanoculture provides a platform to investigate cross-kingdom interactions, which are commonly encountered in biofilm-associated infections in humans, including oral, respiratory, and urinary tract diseases^32^,^33^. Studies of competitive or antagonistic interactions have contributed to the discovery of numerous antibacterial or antifungal compounds^34^,^35^,^36^. While high throughput screening of cells and complex biofilm models exist, there remain significant challenges associated with the analysis of inter-kingdom interactions within localized physical niches, or of the chemical exchanges that occur *in situ* in microcolony-like environments. Spreading the bacteria and fungi on an agar plate to study cross-kingdom interactions also presents several challenges^37^. This method requires significant supplies, limits testing to a few conditions, and may lead to false positives because of the existence of slow-growing cells. Furthermore, this approach fails to address the effects of the physical and chemical fungal-bacterial interactions on cell viability simultaneously and thus it becomes challenging to explore the full potential of the secondary metabolites produced by microorganisms in the presence of pathogens. To overcome these challenges, we chose to spatially sequester one microbial species while investigating how growth dynamics are impacted by chemical exchange (i.e., without physical contacts between cells) and communication with another species across the shell of the nanoculture. Such a situation may be especially important in modeling and studying competition or cooperation among microbes in multispecies communities, factors that are key to identifying new bioactive metabolites and signaling molecules.

**Figure 4:**
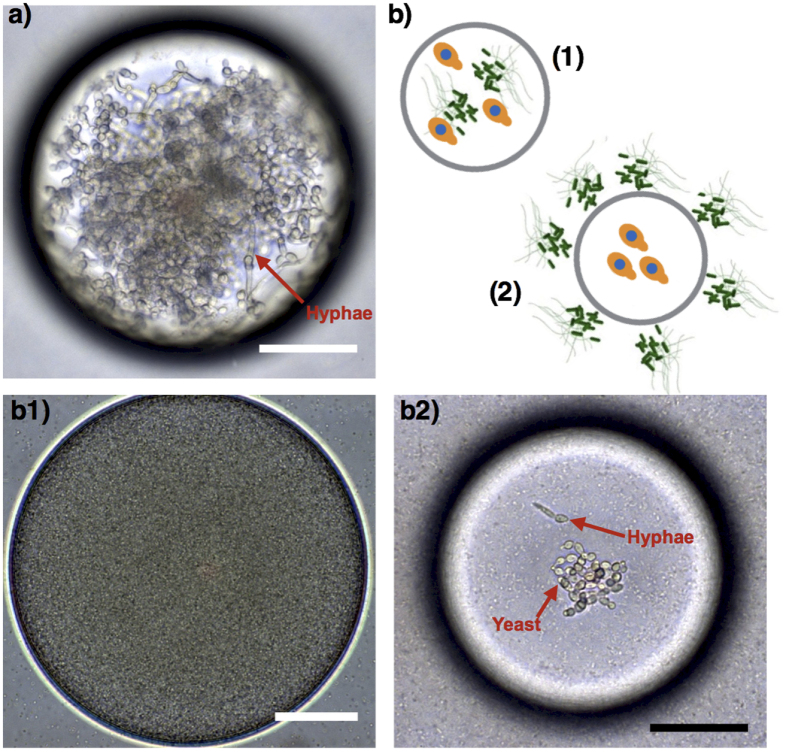
The microbial nanoculture system as a new platform to study cross-kingdom dynamics between *C. albicans* and *P. aeruginosa* PA14. Yeast and hyphae of *C. albicans* were found in nanoculture when the yeast was encapsulated without bacteria **(a)**. However, *C. albicans* were killed in cocultures containing PA14 cells **(b1).** The isolation of *C. albicans* in nanocultures incubated in bacterial culture with PA14 cells shows that growth inhibition of *C. albicans.* Moreover, the cells remained as yeast in the nanocultures when incubated in PA14 culture **(b2)**. (scale bar: 50 μm).

Two distinct conditions were tested to illustrate antagonistic (competitive) cross-kingdom dynamics based on physicochemical interactions or chemical exchange between the fungus *C. albicans* and the bacterium *P. aeruginosa* PA14. In one batch, the bacterial strains were grown together with *C. albicans* as nanocultures (both bacteria and *C. albicans* inside, Fig. 4b1). In the other batch, *C. albicans* were grown alone in the nanocultures, and the *C. albicans* nanocultures were incubated in 24-well plates containing the bacterial culture (bacteria outside of *C. albicans*-containing nanocultures, Fig. 4b2). Control experiments were performed with *C. albicans* grown within nanocultures but without bacteria (i.e., bacteria neither inside nor in the external phase). In these experiments, confluent colonies of yeast and hyphae formed (Fig. 4a). Yeast and hyphae represent the unicellular and vegetative multicellular (or filamentous) states of *C. albicans*, respectively, and can be distinguished from each other by their distinct shapes; yeast are single round cells, whereas hyphae have elongated germ tubes^38^.

Our model of cross-kingdom dynamics not only reproduces the current understanding of direct (physicochemical) and indirect (chemical) *Candida-Pseudomonas* interactions^32^, but also illustrates how spatial isolation influences competition in polymicrobial communities. In both cross-kingdom settings (b1 and b2 in Fig. 4) *C. albicans* failed to form confluent colonies as occurred in the absence of *P. aeruginosa* PA14 cells, suggesting some competition. However, strong antagonistic interaction occurred when PA14 physically interacted with *C. albicans* in the nanoculture (Fig. 4b1), as the bacteria dominated the cocultures. This indicates that *P. aeruginosa* PA14 inhibited the growth of *C. albicans* and killed the co-encapsulated yeasts via chemical and/or physical interactions. Since the yeasts commonly secrete farnesol to decrease bacterial virulence^39^, it is more likely that *C. albicans* killing achieved in the co-encapsulated model was associated with relatively high population density of PA14 cells in the nanocultures, compared to *C. albicans*. Interestingly, a prior study by Hogan *et al*.^40^ reported that physical interaction between *P. aeruginosa* and *C. albicans* in hyphae form provokes killing of the fungus, whereas our work suggest that such interactions could also be detrimental to the yeast, which were eradicated from the nanocultures. However, when the physical interaction between the two cell types is eliminated (Fig. 4b2), a small number of *C. albicans* in the yeast form are observed with a negligible amount of hyphae in the nanoculture (Fig. 4b2). This finding indicates that some metabolic by-products secreted by the PA14 cells, such as the phenazines^41^, could permeate into the fungal nanoculture and trigger a response by *C. albicans*. The yeast, sensing the bacterial chemical attack, may have activated their defense mechanism by repressing hyphal growth. Overall, the versatility of the nanocultures allows us to validate that physical interaction is not necessary to inhibit hyphal growth^32^, a finding that is essential to further explore cross-kingdom microbial pathogenesis and screen for new bioactive molecules.

## Conclusions

Inspired by bacterial microcolonies that form during the initial stages of biofilm development, we have developed microbial nanocultures that artificially sequester cells within a biocompatible and semipermeable PDMS membrane. Using microfluidic technology, we can create hundreds of nanocultures per second, while controlling their size and cell density to mimic microcolony microenvironments. The nanocultures provide a unique opportunity to confine cells within a physical barrier and to investigate their response to chemical, physical and biological stressors that are selectively permeable to PDMS. We used this novel platform to evaluate cell functionality by infusing or entrapping molecular probes including antibiotics and microbial signaling molecules in the nanocultures, which led to the finding that the selectivity of the nanocultures critically depends on the miscibility of these molecules in the shell material (PDMS). The nanoculture system presented in this work is a versatile vehicle to study the short- and long-term multispecies microbial interactions and to address fundamental questions relevant to human drug-resistant infections, and antibiotic discovery. While the selective permeability of the current nanoculture depends on the interactions between PDMS and permeant, our future work will focus on engineering the encapsulation material in order to achieve selectivity toward molecules of interest. This system can lead to a novel platform to help unveil how microbes orchestrate antibiotic resistance, interspecies social behavior, and competitive cross-kingdom interactions.

## Methods

### Microorganisms and growth conditions

*C. albicans* SC 5314^42^, *E. coli* RP437/pRSH109 and *E. coli* RP437*motB580*/pRSH103 (a gift from Dacheng Ren^43^)*, P. aeruginosa* PAO1, PA14 and PA14 Δ*pelA* (a gift from Knut Drescher^16^) were utilized to grow the nanocultures. The cells were cultured in buffered low-molecular weight medium referred to as ultrafiltered tryptone-yeast extract (UFTYE) broth. The UFTYE medium containing 2.5% tryptone, 1.5% yeast extract and 1% glucose had a molecular-weight cut-off of 10 kDa (Millipore). The pH of the medium was adjusted to 5 and 7 to growth *C. albicans* and bacteria, respectively. Briefly, 25 mL of UFTYE medium were introduced in a flask to culture the cells overnight. Then, 50 μL aliquots were introduced in 5 mL UFTYE medium to constitute the inner phase of the microfluidics. The middle phase was encapsulated in PDMS to generate nanocultures, which were incubated at 37 °C in the presence or the absence of 5% CO_2_.

### Fabrication of microfluidic devices

We make use of a glass-capillary microfluidic device with hydrodynamic flow-focusing and coflowing geometry to generate the W/O/W PDMS capsules, as described previously^44^,^45^. Briefly, two circular capillary tubes with inner and outer diameters of 0.58 mm and 1.03 mm (World Precision Instrument Inc.) were tapered to desired diameters using a micropipette puller (P-1000, Sutter Instrument Inc.) and a microforge (Narishige MF-830). The inner diameters of tapered tubes for the injection of a bacteria phase and the collection of capsules were 40 μm and 200 μm, respectively. The outside of the glass capillary tube for inner fluid was hydrophobically functionalized with octadecyltrichlorosilane (OTS). This chemical treatment enhances the wettability of the PDMS outside the capillary tube, and facilitates the formation of capsules. The two tapered capillary tubes were inserted into a square capillary with an inner dimension of 1.05 mm, and the distance between the two tubes for inner bacteria and collection was adjusted to be 120 μm. Subsequently, a transparent epoxy was used to seal the tubes where required.

### Generation of PDMS capsules with bacteria encapsulated

The microfluidic device was mounted on an inverted optical microscope (Eclipse TE 200, Nikon). Then, the three fluid phases were delivered to the microfluidic device through polyethylene tubing (Scientific Commodities) attached to syringes (SGE) that were driven by positive displacement syringe pumps (Harvard Apparatus, PHD 2000 series). The drop formation was monitored with a high-speed camera (Phantom, Vision Research) attached to the inverted microscope. The inner aqueous phase consists of bacteria suspended in culture medium; the middle phase consists PDMS (Sylgard 184, Dow Corning) with 25 wt% low viscosity silicone oil (50cSt, S159-500, Fisher Scientific) and 10 wt% curing agent. The outside phase comprises 5 wt% poly(vinyl alcohol) aqueous solution (PVA, 87–89% hydrolyzed, average MW = 13 000–23 000, Aldrich).

### Staining and imaging of the nanocultures

Acridine orange (Sigma Aldrich), and Filmtracer™ LIVE/DEAD^®^ Biofilm Viability Kit (Thermofisher Scientific) containing Syto9 and propidium iodide were the fluorescent dyes used in this study. To stain cells in the nanocultures, acridine orange was diluted to a final concentration of 15 μM. For Syto9 and propidium iodide, 1.5 μL of dye solution in the kit were added to 1mL of sample. In all cases, staining was performed for 30 min and the samples were gently washed to remove excess dye. The nanocultures were visualized after 24 h of incubation at 37 °C using the Zeiss Axioimager M1 Epifluorescence and Brightfield Microscope (Zeiss, Germany). A LEICA Laser Scanning Confocal Microscope was used to perform fluorescent imaging of nanocultures of *Escherichia coli* RP437/pRSH 109 and *Escherichia coli* RP437 (motB) 580/pRSH, as well as nanocultures stained with fluorescent dyes. In all cases, the samples were collected in the Secureseal^TM^ chamber (Grace Bio-labs) for imaging. The chambers were sealed to prevent micromotion due to drying of the samples.

### Antibiotic susceptibility

To assess the susceptibility of cells within the nanocultures to antibiotics, antibiotics including Amp, Cip, Tet and Tob were introduced in a suspension containing the nanocultures. The antibiotics were added at concentrations comprising between 0–100 μg/mL before the nanocultures were incubated at 37 °C overnight. The next day, the nanocultures were image to evaluate cell growth within the nanocultures. All experiments were performed at least in triplicate.

## Additional Information

**How to cite this article**: Niepa, T. H. R. *et al*. Microbial Nanoculture as an Artificial Microniche. *Sci. Rep.* **6**, 30578; doi: 10.1038/srep30578 (2016).

## Supplementary Material

Supplementary Information
