# Corrigendum: Microbial Nanoculture as an Artificial Microniche

**DOI:** 10.1038/srep42568

**Published:** 2017-02-22

**Authors:** Tagbo H. R. Niepa, Likai Hou, Hongyuan Jiang, Mark Goulian, Hyun Koo, Kathleen J. Stebe, Daeyeon Lee

Scientific Reports 6 Article number: 3057810.1038/srep30578; published online: 08 01 2016; updated on: 02 22 2017

The Acknowledgements section in this Article is incomplete.

“This work was supported by NSF Grant No. DMR-1120901 (Penn MRSEC) and PIRE-1545884. T.H.R.N. was supported by the Postdoctoral Fellowship for Academic Diversity Program (University of Pennsylvania) and the Consortium for Ocean Leadership, Inc. (Prime award #SA15-19). H.K. was supported by NIH/NIDCR grants R01DE018023 and R01DE025220. L.H. and H.J. were supported by Natural Science Foundation of China (NSFC) grant #11372093 and China Scholarship Council (CSC) Grant #201306120117. We also thank Dacheng Ren (Syracuse University) for providing the *E. coli* strains; Knut Drescher (Max Planck Institute for Terrestrial Microbiology, Germany) for *P. aeruginosa* PA14 strains”.

should read:

“This research was made possible in part by a grant from The Gulf of Mexico Research Initiative, NSF Grant No. DMR-1120901 (Penn MRSEC), and PIRE-1545884. T.H.R. N. was supported by the Postdoctoral Fellowship for Academic Diversity Program (University of Pennsylvania). H.K. was supported by NIH/NIDCR grants R01DE018023 and R01DE025220. L.H. and H.J. were supported by Natural Science Foundation of China (NSFC) grant #11372093 and China Scholarship Council (CSC) Grant #201306120117. We also thank Dacheng Ren (Syracuse University) for providing the *E. coli* strains; Knut Drescher (Max Planck Institute for Terrestrial Microbiology, Germany) for *P. aeruginosa* PA14 strains. Data are publicly available through the Gulf of Mexico Research Initiative Information & Data Cooperative (GRIIDC) at https://data.gulfresearchinitiative.org (doi:10.7266/N78G8HQC, doi:10.7266/N7D50K1B)”.

