# Supplementary material for: Microbial Nanoculture as an Artificial Microniche
Source: Sci Rep. 2016 Aug 1;6:30578. doi: 10.1038/srep30578 (PMC4967889; doi:10.1038/srep30578)
Supplement: Supplementary Information [file srep30578-s1.pdf]

Supplementary Information

Microbial Nanoculture as an Artificial Microniche

Tagbo H. R. Niepa[1], Likai Hou[1,2], Hongyuan Jiang[2], Mark Goulian[3], Hyun Koo[4], Kathleen J. Stebe*[1] and Daeyeon Lee*[1]

[1]Department of Chemical and Biomolecular Engineering, University of Pennsylvania, Philadelphia, PA 19104, USA

[2]School of Mechatronics Engineering, Harbin Institute of Technology, Harbin 150001, China.

[3]Department of Biology, University of Pennsylvania, Philadelphia, PA 19104, USA,

[4]Department of Orthodontics, Pediatric Dentistry and Community Oral Health Divisions, University of Pennsylvania, PA19104, USA.

*Corresponding authors contributed equally to the work

Kathleen Stebe: kstebe@seas.upenn.edu

Daeyeon Lee: daeyeon@seas.upenn.edu

1- Characterization of PDMS Nanoculture systems.

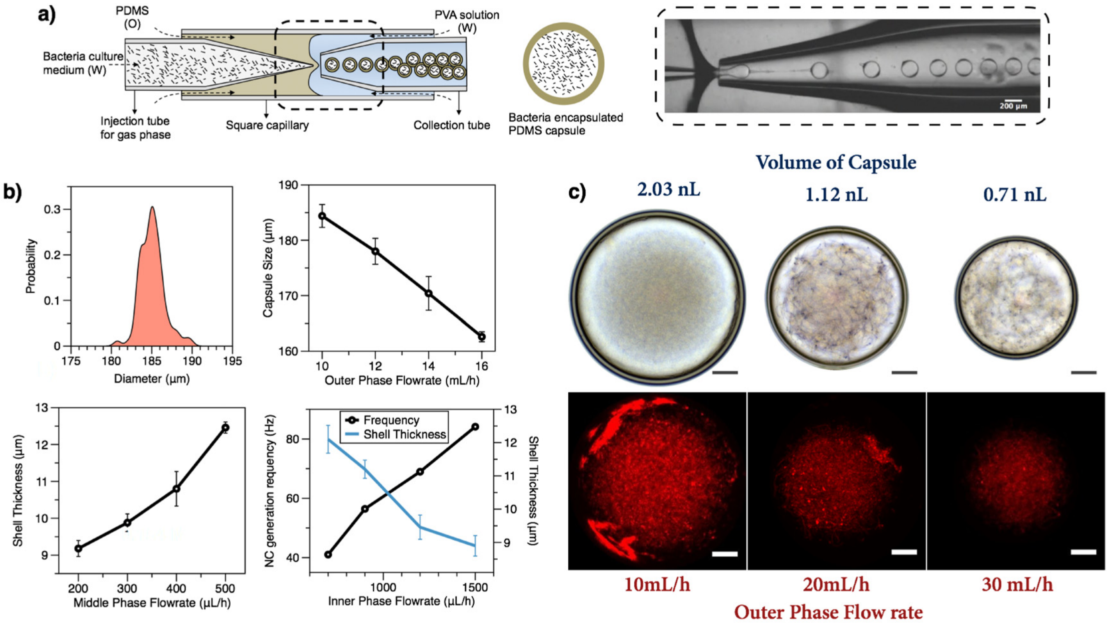

Fig S1. (a) Microfluidic device composed of two circular capillary tubes, coaxially aligned and opposed to each other within a square glass capillary. The circular capillary tubes have been thermally pulled to have tapered openings of 40 µm and 200 µm in diameter and were spaced from each other by 120 µm. Such a configuration permitted the inflow of the inner (bacterial suspensions in growth medium), middle (PDMS containing 25% of silicon oil and 10% of curing agent), and outer (5% PVA solution) phases, as well as the outflow and collection of highly monodisperse water-in-oil-in-water (W/O/W) double emulsions. The size, shell thickness and generation frequency of the double emulsions were controlled by varying the flow rates of the three phases. **(b)** While the increase of the outer phase flow rate resulted in the decrease of the droplet size, the thickness of the capsule shell was increased with the flow rate of the middle phase. **(c)** Similarly, the generation frequency of double emulsions increased with the flow rate of both the inner and outer phases. For instance, setting the flow rate of the inner, middle

and outer phases to 250 μL/h, 730 μL/h and 10,000 μL/h, respectively, yielded double emulsion droplets with an internal volume of 2 nL a rate of 92 capsules per sec. However, doubling and tripling the outer flow rate led to the formation of double emulsions with 1.12 nL and 0.71nL internal volume, at a frequency of 184.0 and 234.5 capsules per sec, respectively.

2- Semi-permeability of PDMS Nanoculture to antibiotics.

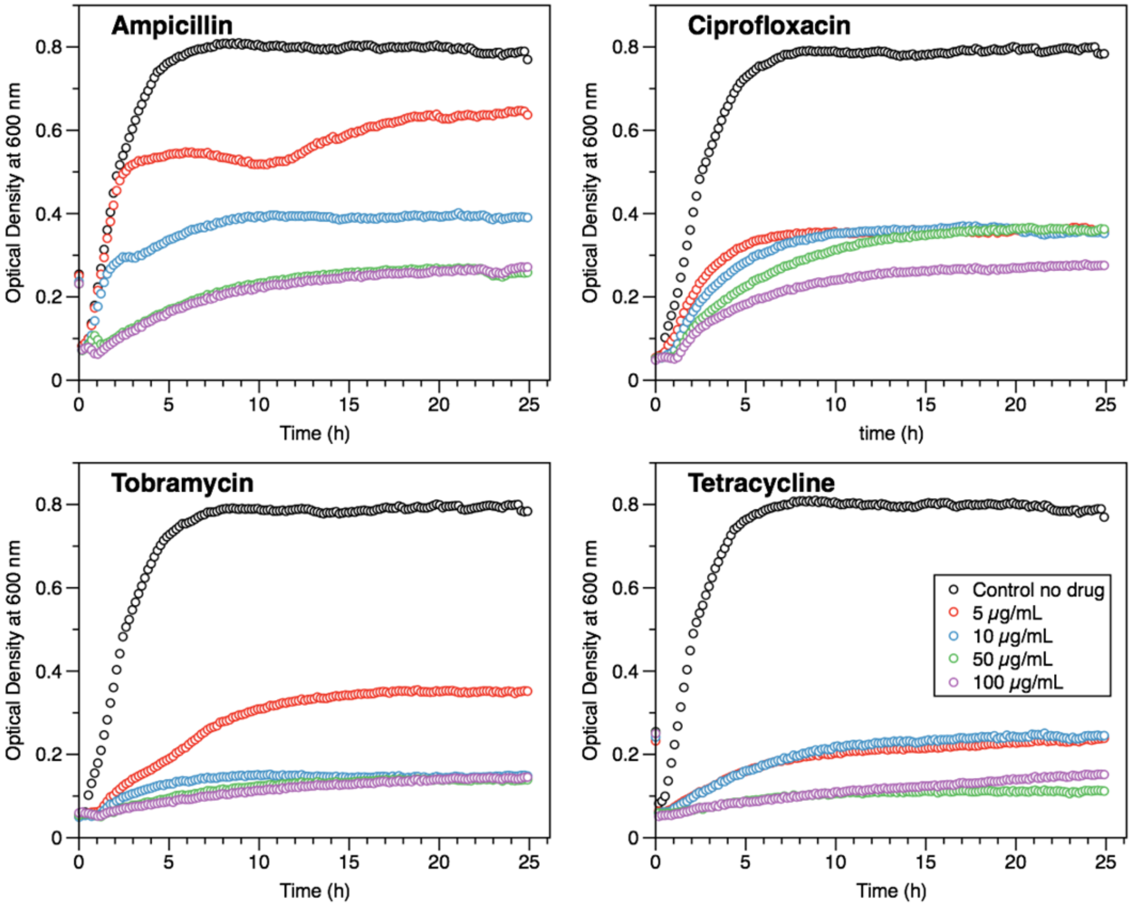

Fig S2a. Antibiotic susceptibility of bacteria under a normal culture condition. To demonstrate that *E coli* RP437 cells were susceptible to the four antibiotics used in this study, the cells were grown in 200 μL LB with or without Amp, Cip, Tet and Tob. As shown in the growth curves, the antibiotics inhibited the growth of *E. coli* RP437 cells. While *E. coli* RP437 growth in LB reached an optical density (OD_{600}) of 0.8 in 6 h and remained stationary, Amp had a dose-dependent inhibitory activity. Also, the presence of 5 μg/mL Cip, Tob, and Tet reduced *E. coli* growth rate by half, and further decreased at higher concentrations. Thus, the inability of Tet as well as Tob to kill the cells in the nanocultures confirmed that these molecules were impermeable to PDMS, in contrast to Cip and Amp that exhibited strong antibacterial activities in the nanocultures.

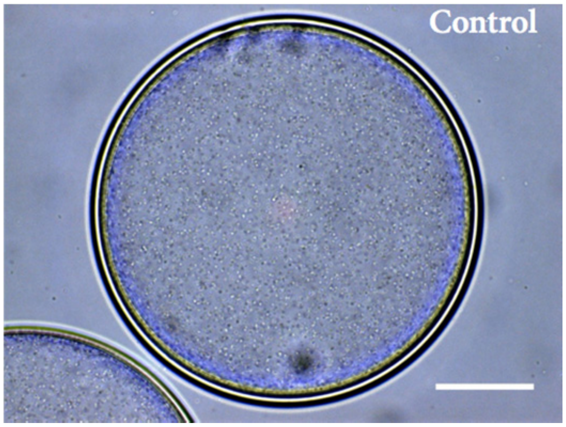
Control

(1)

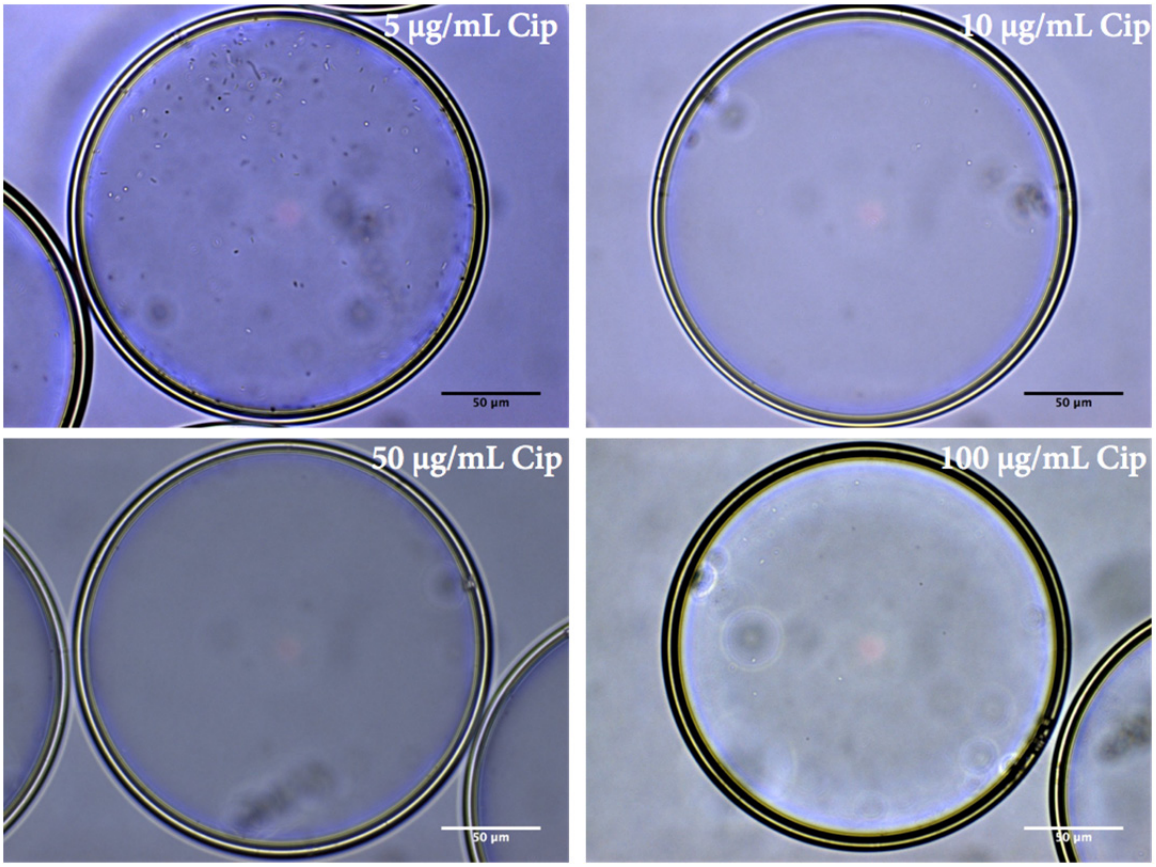
5 μg/mL Cip
50 μm
10 μg/mL Cip
50 μm
50 μg/mL Cip
50 μm
100 μg/mL Cip
50 μm

(2)

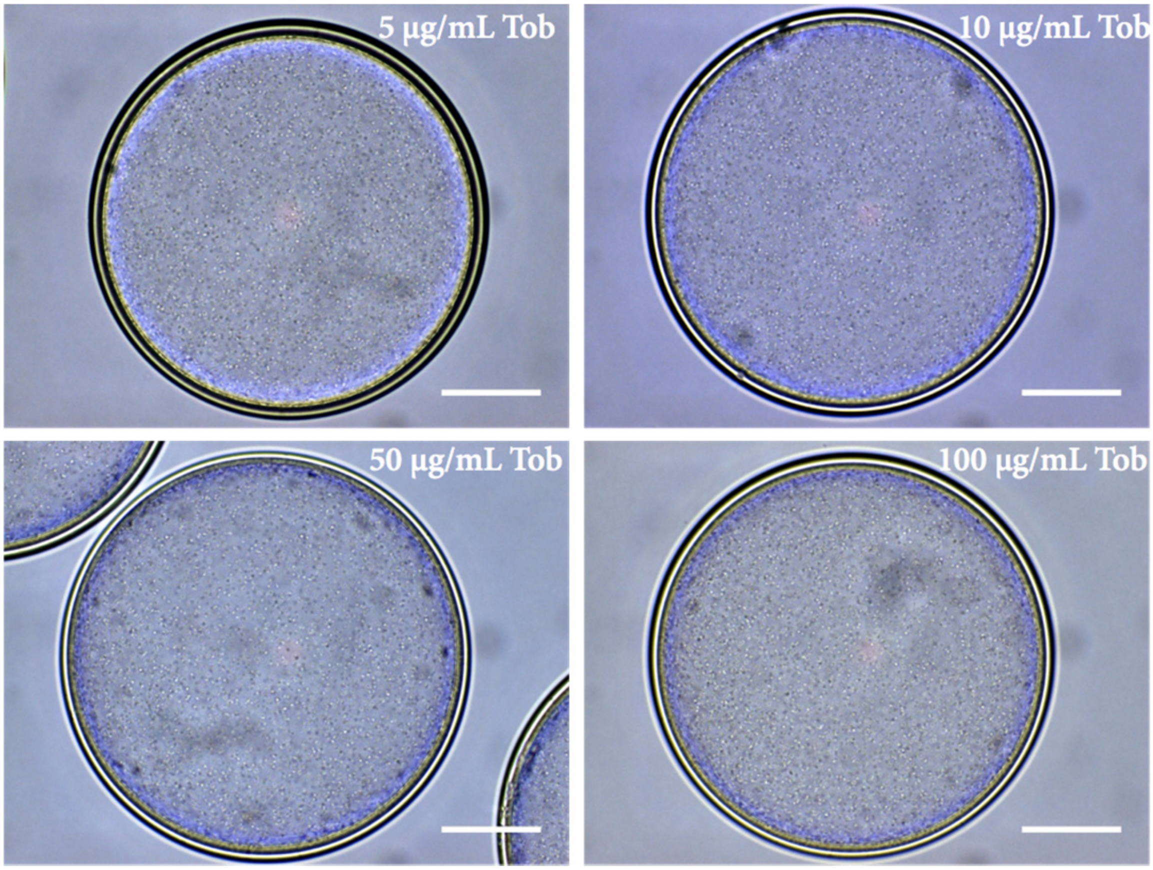

(3)

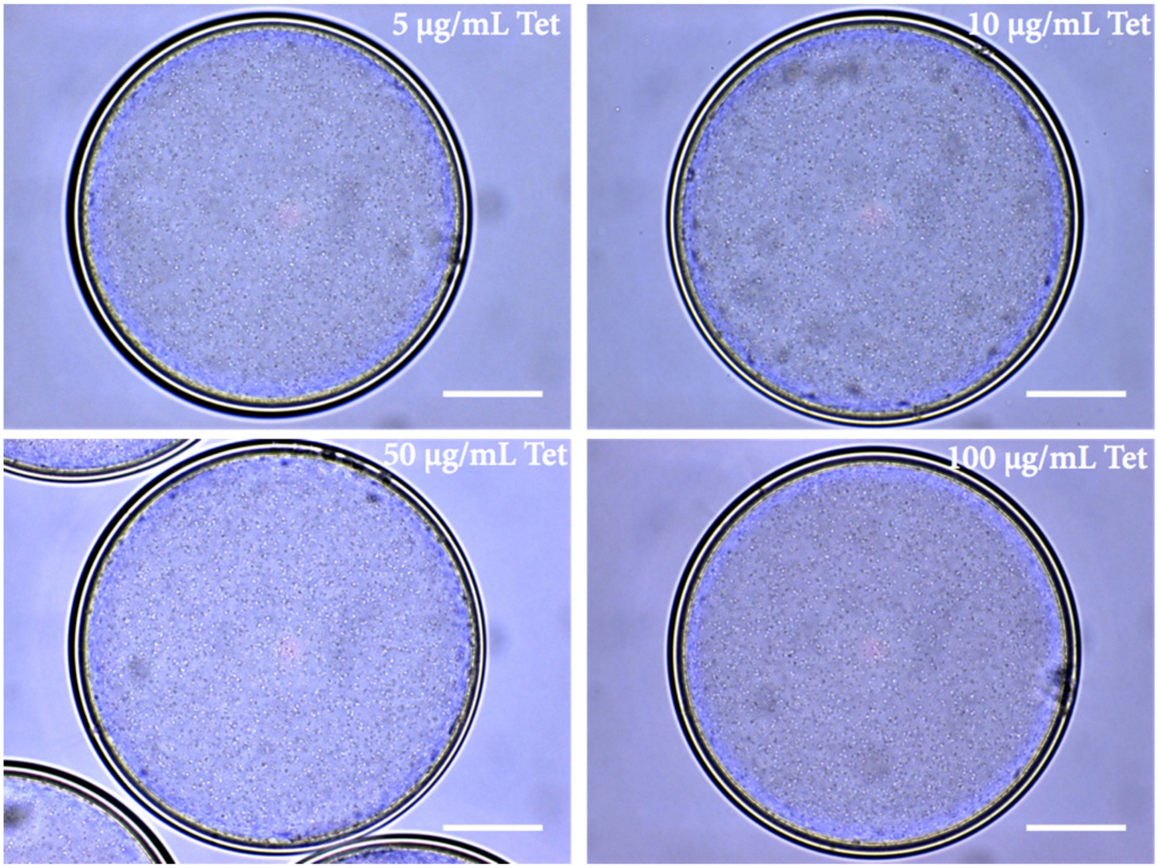

(4)

Fig S2b. Permeability of the PDMS nanoculture to antibiotics. *P. aeruginosa* cells were encapsulated and the nanocultures were collected in a solution of 0.85% NaCl. Antibiotics including β-lactam ampicillin (Amp), aminoglycoside tobramycin (Tob), fluoroquinolone ciprofloxacin (Cip), and tetracycline (Tet) were introduced in the external phase, the 0.85% NaCl collection solution, at concentrations between 0-100 μg/mL, and the nanocultures were incubated for 24 h at 37°C. Compared to the untreated control **(1)**, the growth of the PA14 cells treated with Cip **(2)** was inhibited during the 24h incubation period. However, the inability of Tob **(3)**, and Tet **(4)** to cross PDMS nanoculture is clearly demonstrated by the growth of the PA14 cells in the nanoculture incubated in the presence of 0-100 μg/mL Tob and Tet.

3- Size of the molecules crossing the shell of the nanocultures.

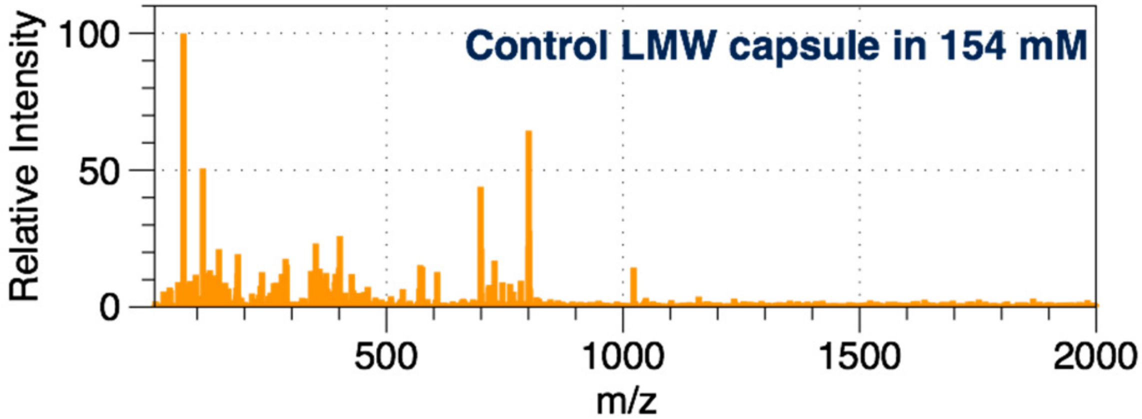

Fig S3. Size-exclusion through PDMS nanocultures. Nanocultures of *P. aeruginosa* PAO1 was generated and collected in saline solution containing 154, 400, 600, 800 and 1000mM NaCl. The nanocultures were then grown overnight at 37°C. Two control samples including sterile UFTYE medium and 154mM NaCl solution as inner phase, were generated, collected in 154mM NaCl solution, and incubated at 37°C. The supernatant of the nanocultures and the sterile capsules were collected to perform HPLC analysis to determine the molecular weight of elements released after 24h incubation. Shimadzu high-performance liquid chromatograph/mass spectrometer (Shimadzu, Columbia MD) was used for LC-MS analysis. To perform HPLC analysis, 20 µL samples was injected in a medium sized PRP1 column, at a rate of 1mL/min. HPLC grade acetonitrile and water were used as the stationary and the mobile phases, respectively. The stationary phase was run at a gradient starting at 5% (vs. 95% mobile phase) to 95% (vs. 5% mobile phase) in 15 min, and maintained at 95% for the remaining 15 min. Eluted elements were analyzed using LCMS 2010EV (Shimadzu, Columbia MD). The results demonstrated that the encapsulation of LB medium in the absence of cells lead to the release of molecules of molecular weights ranging from 0 to 1000 g/mol.

4- Flory–Huggins interaction parameter: Miscibility of selected molecules in PDMS.

The Flory-Huggins interaction parameter (χ) for a molecule and a polymer can be determined using the following relationship:

$$\chi = V(\delta_d - \delta_p)^2/RT$$

where V represents the molar volume of the segment, δ_d and δ_d the Hilderbrand solubility parameters of the molecule and the polymer, respectively, R the gas constant and T the absolute temperature. To estimate the chi parameters, we use the Hansen solubility parameters, which account for nonpolar (δ_{di}), polar (δ_{pi}) and hydrogen bonding (δ_{hi}) interactions between the solvent and the polymer. The shell of the nanoculture in our system is a mixture of PDMS (Silgard 184) and silicon oil. Hildebrand solubility parameters of silicon oil and PDMS are 15.142(MPa$^{1/2}$)[1] and 18.933(MPa$^{1/2}$)[2], respectively. Hansen solubility parameters of some of the molecules we used in this study have been reported and are summarized in the following table.

	δ_d (MPa$^{1/2}$)	V (cm^3/mol)	χ w/ Silicone Oil	χ w/ PDMS	Permeability
CO_2 [3]	17.4	39.13	0.08	0.04	Yes
O_2 [4]	14.7	22.6	0.00	0.16	Yes
Ampicillin[5]	25.78	224.3	9.85	4.08	Yes
Ciprofloxacin[6]	26.22	211.8	10.09	4.37	Yes
Water[7]	47.8	18.02	7.46	5.83	Yes
Glucose[5]	41.72	92.9	25.46	18.72	No
Tetracycline[6]	34.53	235.4	34.33	22.22	No
Sucrose[5]	41.31	159.5	42.37	30.99	No

References

1 Kurita, H., Tateishi, T., Suzuki, T. & Higo, N. (Google Patents, 2009).

2 Su, X., Shi, B. & Wang, L. Investigation on Three-Dimensional Solubility Parameters for Explanation and Prediction of Swelling Degree of Polydimethylsiloxane Pervaporation Membranes. *J. Macromol. Sci. B* **54**, 1248-1258 (2015).

3 Williams, L. L., Rubin, J. B. & Edwards, H. Calculation of Hansen solubility parameter values for a range of pressure and temperature conditions, including the supercritical fluid region. *Ind. Eng. Chem. Res.* **43**, 4967-4972 (2004).

4 Hansen, C. M. *Hansen solubility parameters: a user's handbook*. (CRC press, 2007).

5 Thakral, S. & Thakral, N. K. Prediction of drug–polymer miscibility through the use of solubility parameter based Flory–Huggins interaction parameter and the experimental validation: PEG as model polymer. *J. Pharm. Sci.* **102**, 2254-2263 (2013).

6 Breitkreutz, J. Prediction of intestinal drug absorption properties by three-dimensional solubility parameters. *Pharm. Res.* **15**, 1370-1375 (1998).

7 Deshmukh, S. & Li, K. Effect of ethanol composition in water coagulation bath on morphology of PVDF hollow fibre membranes. *J. Membr. Sci.* **150**, 75-85 (1998).
